# Supplementary material for: Identification of a NACC1-Regulated Gene Signature Implicated in the Features of Triple-Negative Breast Cancer
Source: Biomedicines. 2023 Apr 20;11(4):1223. doi: 10.3390/biomedicines11041223 (PMC10136325; doi:10.3390/biomedicines11041223)
Supplement: Supplementary file 1 [file biomedicines-11-01223-s001.zip › biomedicines-2274842-supplementary.pdf]

Supplementary Figures and legends.

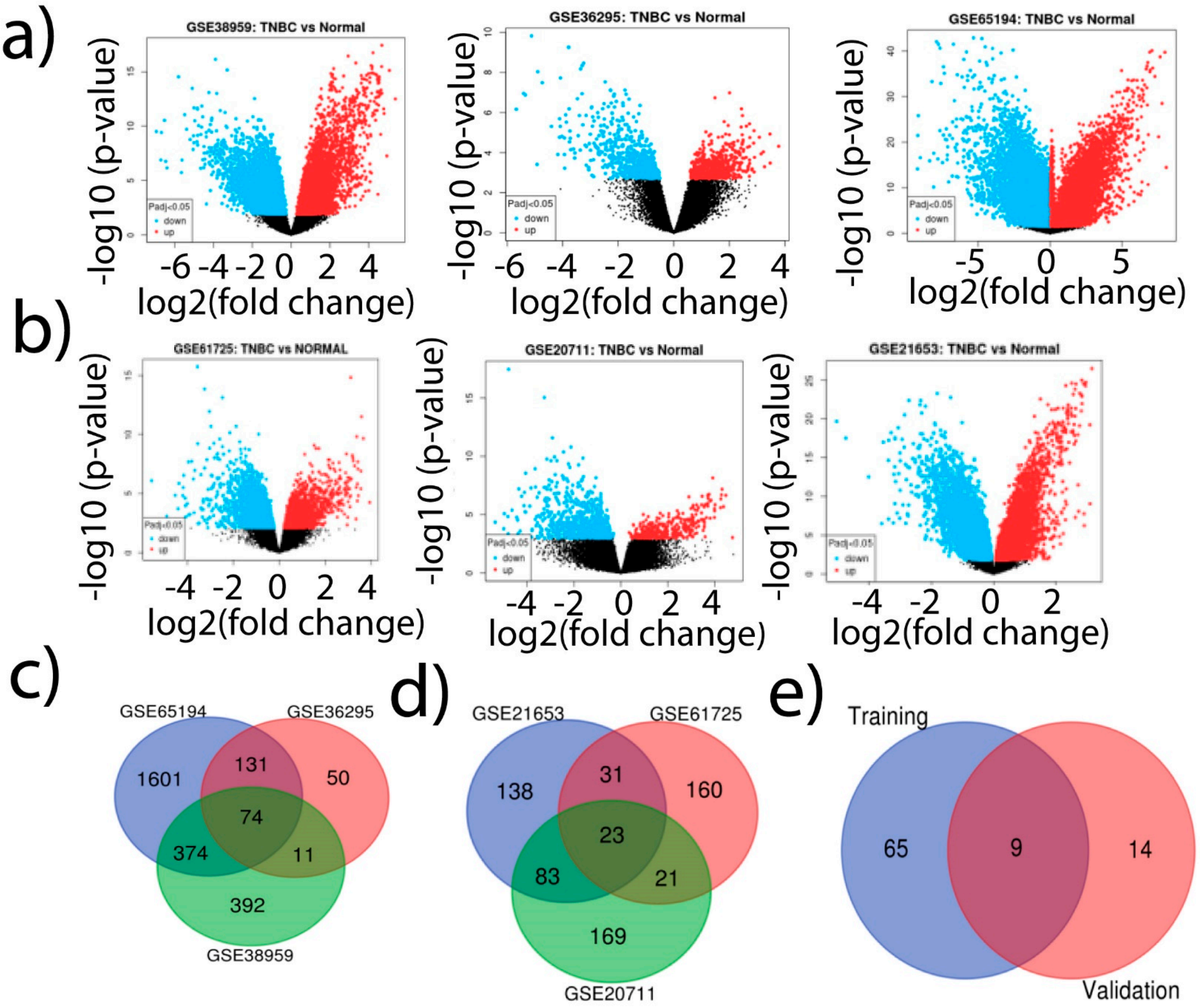

S. Figure 1. Identification of the common upregulated and downregulated genes in TNBC. (**a**) Training group datasets volcanos; (**b**) Validation group datasets volcanos. Color code: Blue represents downregulated genes; red represents the upregulated genes; (**c**) The common downregulated genes in the training group; (**d**) The common downregulated genes in the validation group; (**e**) The common downregulated genes in the training and validation groups.

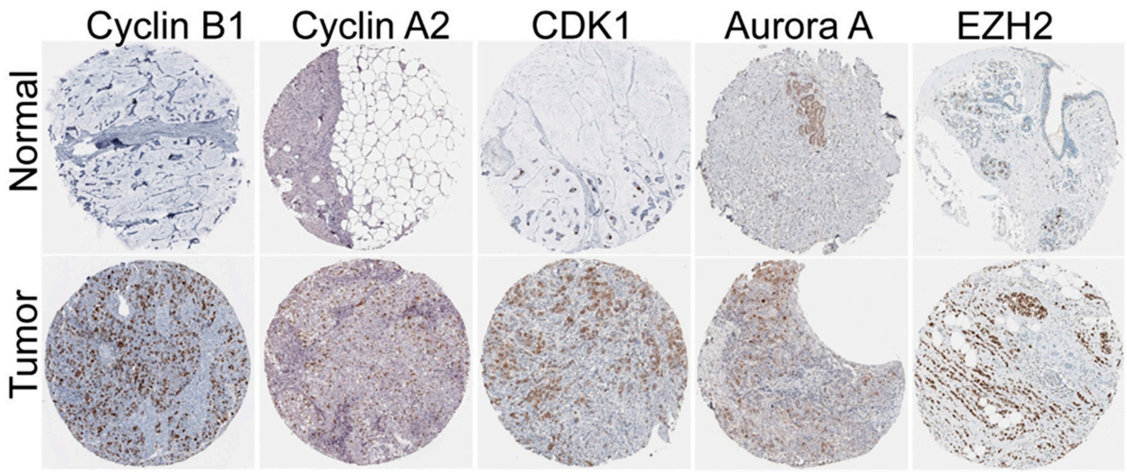

S. Figure 2. Expression of the stemness-associated proteins in BC vs. normal tissues samples from the human protein atlas.

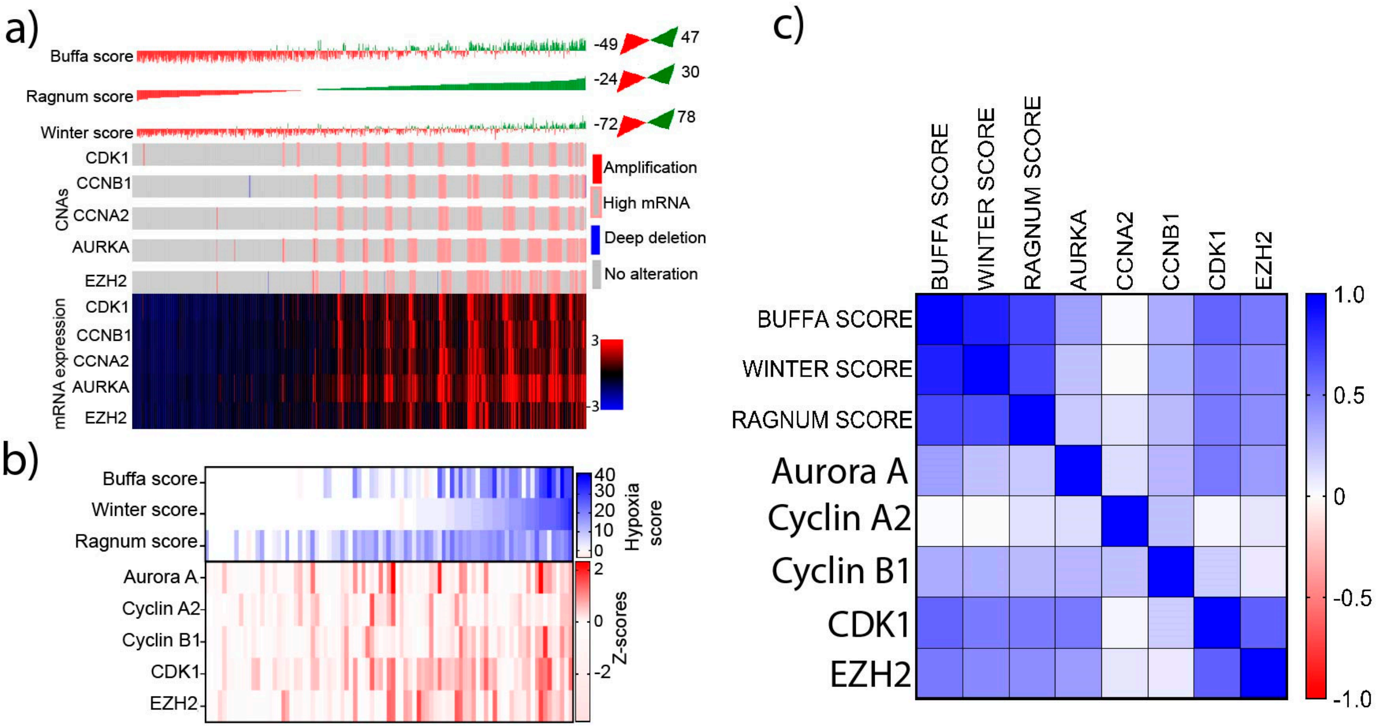

S. Figure 3. Correlation of the signature genes expression with tumor hypoxic status. (**a**) CNAs and mRNA expression profiles of signature genes with accompanying changes of hypoxia status in TCGA samples. Green color code represents high hypoxia and red color code represents low hypoxia; (**b**) Protein expressions of the signature genes in CPTAC samples vs. hypoxia conditions; (**c**) Correlation of protein expressions of the signature genes with hypoxia signature scores.

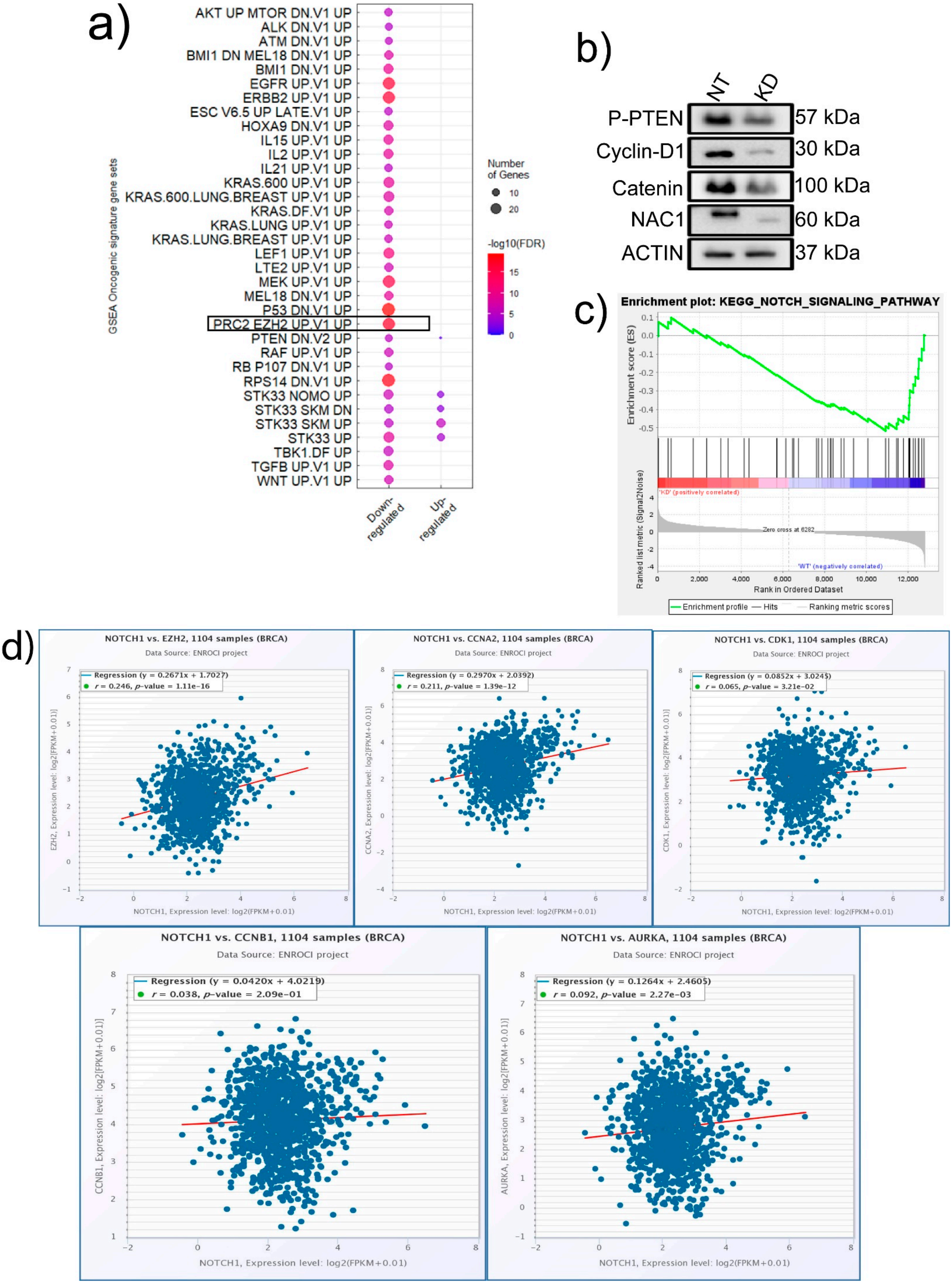
a)
GSEA Oncogenic signature gene sets
AKT UP MTOR DN.V1 UP
ALK DN.V1 UP
ATM DN.V1 UP
BMI1 DN MEL18 DN.V1 UP
BMI1 DN.V1 UP
EGFR UP.V1 UP
ERBB2 UP.V1 UP
ESC V6.5 UP LATE.V1 UP
HOXA9 DN.V1 UP
IL15 UP.V1 UP
IL2 UP.V1 UP
IL21 UP.V1 UP
KRAS.600 UP.V1 UP
KRAS.600.LUNG.BREAST UP.V1 UP
KRAS.DF.V1 UP
KRAS.LUNG UP.V1 UP
KRAS.LUNG.BREAST UP.V1 UP
LEF1 UP.V1 UP
LTE2 UP.V1 UP
MEK UP.V1 UP
MEL18 DN.V1 UP
P53 DN.V1 UP
PRC2 EZH2 UP.V1 UP
PTEN DN.V2 UP
RAF UP.V1 UP
RB P107 DN.V1 UP
RPS14 DN.V1 UP
STK33 NOMO UP
STK33 SKM DN
STK33 SKM UP
STK33 UP
TBK1.DF UP
TGFB UP.V1 UP
WNT UP.V1 UP
Down-regulated
Up-regulated
Number of Genes
10
20
-log10(FDR)
15
10
5
0
b)
NT
KD
P-PTEN
57 kDa
Cyclin-D1
30 kDa
Catenin
100 kDa
NAC1
60 kDa
ACTIN
37 kDa
c)
Enrichment plot: KEGG_NOTCH_SIGNALING_PATHWAY
Enrichment score (ES)
'KD' (positively correlated)
Zero cross at 6282
'WT' (negatively correlated)
Ranked list metric (Signal2Noise)
Rank in Ordered Dataset
Enrichment profile
Hits
Ranking metric scores
d)
NOTCH1 vs. EZH2, 1104 samples (BRCA)
Data Source: ENROCI project
Regression (y = 0.2671x + 1.7027)
r = 0.246, p-value = 1.11e-16
EZH2, Expression level: log2[FPKM+0.01)]
NOTCH1, Expression level: log2(FPKM+0.01)
NOTCH1 vs. CCNA2, 1104 samples (BRCA)
Data Source: ENROCI project
Regression (y = 0.2970x + 2.0392)
r = 0.211, p-value = 1.39e-12
CCNA2, Expression level: log2[FPKM+0.01)]
NOTCH1, Expression level: log2(FPKM+0.01)
NOTCH1 vs. CDK1, 1104 samples (BRCA)
Data Source: ENROCI project
Regression (y = 0.0852x + 3.0245)
r = 0.065, p-value = 3.21e-02
CDK1, Expression level: log2[FPKM+0.01)]
NOTCH1, Expression level: log2(FPKM+0.01)
NOTCH1 vs. CCNB1, 1104 samples (BRCA)
Data Source: ENROCI project
Regression (y = 0.0420x + 4.0219)
r = 0.038, p-value = 2.09e-01
CCNB1, Expression level: log2[FPKM+0.01)]
NOTCH1, Expression level: log2(FPKM+0.01)
NOTCH1 vs. AURKA, 1104 samples (BRCA)
Data Source: ENROCI project
Regression (y = 0.1264x + 2.4605)
r = 0.092, p-value = 2.27e-03
AURKA, Expression level: log2[FPKM+0.01)]
NOTCH1, Expression level: log2(FPKM+0.01)

S. Figure 4. Analysis of the RNA seq data from the NACC1-depleted tumor cells and *in vitro* validation of affected pathways. (**a**) GSEA oncogenic signature analysis of RNA-seq data from tumor cells with NAC1 depletion vs. tumor cells without NAC1 depletion; (**b**) Western blot analysis to validate the changes of the related pathways in MDA-MB-231 cells with NAC1 depletion. **(c)** KEGG NOTCH pathway PGSEA enrichment from NAC1 depleted cells RNA seq data. **(d)** Analysis of the NOTCH pathway correlation with the identified signature genes.

S. Table 1. Interaction scores of the signature genes

**Interaction scores of the signature genes**		
**node1**	**node2**	**score**
CDK1	CCNB1	0.999
CDK1	CCNA2	0.999
CCNB1	CDK1	0.999
CCNA2	CDK1	0.999
CCNB1	AURKA	0.986
CCNA2	AURKA	0.986
AURKA	CCNA2	0.986
AURKA	CCNB1	0.986
EZH2	CDK1	0.98
CDK1	EZH2	0.98
CCNB1	CCNA2	0.976
CCNA2	CCNB1	0.976
CDK1	AURKA	0.975
AURKA	CDK1	0.975
EZH2	CCNA2	0.76
CCNA2	EZH2	0.76
EZH2	CCNB1	0.654
CCNB1	EZH2	0.654
EZH2	AURKA	0.644
AURKA	EZH2	0.644

S. Table 2. Validation of the signature genes

Term description	observed gene count	background gene count	strength	false discovery rate	matching SGs in network
Histone phosphorylation	4	31	2.7	4.69E-07	AURKA, CCNB1, CCNA2, CDK1
Regeneration	5	157	2.1	4.69E-07	AURKA, CCNB1, CCNA2, EZH2, CDK1
Animal organ regeneration	4	75	2.32	5.25E-06	AURKA, CCNA2, EZH2, CDK1
Histone modification	5	351	1.75	6.22E-06	AURKA, CCNB1, CCNA2, EZH2, CDK1
G2/M transition of mitotic cell cycle	4	134	2.07	1.89E-05	AURKA, CCNB1, CCNA2, CDK1
Positive regulation of cell cycle process	4	294	1.73	0.0002	AURKA, CCNB1, EZH2, CDK1
DNA damage response	3	59	2.3	0.00022	AURKA, CCNB1, CDK1
Cell cycle process	5	976	1.3	0.00022	AURKA, CCNB1, CCNA2, EZH2, CDK1

S. Table 3. WIKIpathway analysis of the five signature genes.

Term description	observed gene count	background gene count	strength	false discovery rate	matching SGs in network
Retinoblastoma gene in cancer	3	87	2.13	0.00063	CCNB1, CCNA2, CDK1
Cell cycle	3	119	1.99	0.00079	CCNB1, CCNA2, CDK1
ATM signaling pathway	2	39	2.3	0.0096	CCNB1, CDK1
AMP-activated protein kinase (AMPK) signaling	2	68	2.06	0.0189	CCNB1, CCNA2
miRNA regulation of DNA damage response	2	70	2.05	0.0189	CCNB1, CDK1
G1 to S cell cycle control	2	64	2.09	0.0189	CCNB1, CDK1
DNA damage response	2	67	2.07	0.0189	CCNB1, CDK1
